# Simple and high-containment lung-on-chip model for studying respiratory viral infections using human primary lung cells

**DOI:** 10.1016/j.mtbio.2025.102316

**Published:** 2025-09-15

**Authors:** David Barata, Sem Koornneef, Francesca Giacomini, Zeinab Niloofar Tahmasebi Birgani, Jiangrong Zhou, Pengfei Li, Robbert J. Rottier, Roman K. Truckenmüller

**Affiliations:** a MERLN Institute for Technology-Inspired Regenerative Medicine, Maastricht University, Maastricht, the Netherlands; b Department of Pediatric Surgery/Cell and Developmental Biology, Erasmus MC-University Medical Center, Rotterdam, the Netherlands; c Department of Gastroenterology and Hepatology, Erasmus MC-University Medical Center, Rotterdam, the Netherlands

**Keywords:** Lung on chip, Airway, Human primary bronchial epithelial cells, (Human) coronaviruses, Viral infection

## Abstract

Airborne respiratory viruses, such as coronaviruses and influenza, pose major threats to public health and the economy, as highlighted by the COVID-19 pandemic. Preclinical research is hindered by models that poorly mimic human tissue structure and function, often relying on immortalized cell lines and low-throughput animal studies. This limits accurate prediction of disease mechanisms, drug effects, and target suitability. Here, we report a custom-engineered, passive-flow, high-containment chip for culturing human primary bronchial epithelial cells (hPBECs) at air-liquid interface (ALI) on a large-area membrane. The dual-chamber microfluidic chip, separated by a horizontal support membrane, is enclosed in a 35 mm sealed Petri dish, enabling safe use in standard incubators without leakage or biosafety concerns. The platform supports high-resolution in-situ imaging, apical viral infection, and retrieval of cells and secretions (e.g., mucus, viral lysate) for molecular analysis. We demonstrate robust infection and replication of human coronavirus NL63 (HCoV-NL63) in differentiated hPBECs cultured up to 4 weeks at ALI. Epithelial differentiation was confirmed by immunofluorescence (e.g., ciliated cells), and infection kinetics were monitored by RT-qPCR over 7 days. The interferon-based immune response showed increased activity, with upregulation of viral response pathways (e.g., replication, inflammation, immunoregulation), and consistent activation across donors (e.g., ISG15, IFIT1). Collectively, we present a reproducible, small-scale chip model that enables high-containment *in vitro* studies of respiratory viruses and their effects on human airway epithelia.

## Graphical abstract

**Figure:**
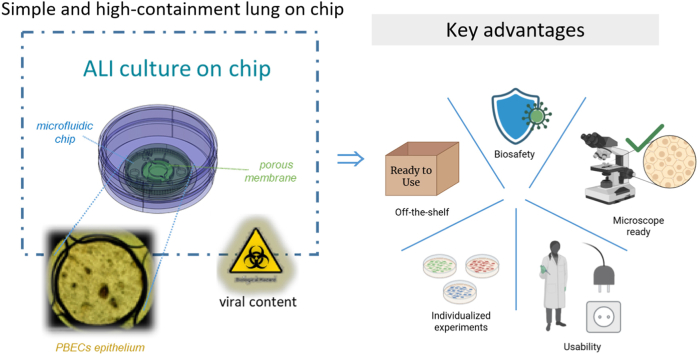

## 1 Introduction

The last 30 years showed a decrease in the prevalence of respiratory tract infections, but airway infections still contribute to a large sum of medical costs each year, e.g., 21 billion USD in the US alone [1]. A joint report by the World Health Organization (WHO) and the European Respiratory Society (ERS) estimates that in Europe, annual productivity losses due to chronic respiratory diseases among the population aged 30–74 exceed $20.7 billion [2]. The recent COVID-19 pandemic emphasized once again the importance of good prevention strategies and for adequate responses to viral airway infections. Human coronaviruses (HCoV), including HCoV-NL63, HCoV-229E, HCoV-OC43, and HCoV-HKU1, are endemic and circulate regularly within the human population. They typically cause seasonal outbreaks, often peaking during colder months [[3], [4], [5]]. To accelerate the development of new targeted drugs against new coronavirus variants and future viral outbreaks, we need physiologically relevant *in vitro* models that capture patient's heterogeneity pool and demographics diversity [[6], [7], [8]]. Unfortunately, many standard cell lines, such as the commonly used VeroE6, lack expression of angiotensin-converting enzyme 2 (ACE2), a key receptor for both SARS-CoV-2 and HCoV-NL63 infection [8,9]. *In vitro* studies of COVID-19 pathology have recently demonstrated the value of using human primary bronchial epithelial cells (hPBECs) [10]. The expression of the ACE2 receptor is influenced by age, sex, smoking status, and pathogenic conditions [[11], [12], [13], [14]]. Following infection, lung cells initiate the production of anti-inflammatory agents as part of the immune response [15,16]. Additionally, innate immunity genes, like STAT1 [17], ISG15 [17], and MX1 [18], were also found to be upregulated during viral infections, both in patients and in *in vitro* cultures. These genes provide a reliable readout for assessing differential responses among donors. hPBECs, which are isolated from patients' surgical resections, can be expanded and differentiated *in vitro* to recapitulate a mucociliary architecture, thereby enabling the assessment of differential responses among different donors [16,19]. PBECs have been useful to support models to study mechanistic studies on defense against respiratory viruses [16,[20], [21], [22], [23]]. Building on this, selecting the appropriate cell type in a biobank is critical for identifying effective treatments, particularly in the context of current and emerging viral threats, where host-pathogen interactions can vary significantly between cell types [24,25].

In addition to the selected cell types, infectious diseases can be investigated using a range of *in vitro* cell culture systems, including submerged, three-dimensional (3D) cultures (e.g., spheroids, organoids, scaffolds), air-liquid interface (10.13039/501100010665ALI) cultures on permeable supports (i.e. cell culture inserts) and, more recently, organ-on-chip platforms (OoCs) [[26], [27], [28], [29]]. Lung-on-chip (LoCs) models have proven to provide differentiated human bronchial airway epithelium to model viral infections, such as influenza and SARS-CoV-2, showing strain-dependent virulence, cytokine production, and the recruitment of circulating immune cells [30]. This rich analysis has contributed to rapid screening for the repurposing of antivirals and discovering new drug candidates to study infection kinetics [[30], [31], [32]]. Generally, organ-on-chip systems house microtissues within a microfluidic environment and utilize perfusion to ensure consistent nutrient delivery and the removal of metabolic waste. This dynamic environment also provides essential physiological signals - from mechanical cues like shear stress to biochemical cues like local gradients - that are critical for driving proper cell differentiation and function [33,34]. Conventionally, this is achieved with complex external pump and tubing systems that are expensive, laborious, and require specialized training. Critically, these setups are ill-suited for high-containment (e.g., BSL-3) viral studies, as their multiple connection points pose a significant risk of leaks and make decontamination difficult. While OoCs platforms provide a powerful, animal-free alternative for investigating human organ physiology and disease, their operational complexity and high cost remain significant barriers [35,36]. Hence, these factors limit their widespread adoption, particularly within most respiratory medicine and virology laboratories. These difficulties may prevent many experts from uncovering key mechanisms of viral pathogens by using microphysiological systems as *in vitro* models, such as LoCs.

Although many *in vitro* studies of the airway are performed with an apical-only configuration [15,29,37,38], different approaches have been developed to expose both the basolateral and apical sides during epithelial differentiation, including culture inserts [39] and on-chip configurations [30,40,41]. The introduction of an endothelial barrier in these systems highlights the importance of tissue vascularization and its role in mediating exchanges between the air and the circulatory system. By incorporating this complexity, OoC models, provide relevant data for evaluating drug permeability, inflammatory processes, and vascular integrity under stress [30,38,[41], [42], [43]]. Despite their potential, limitations arise from technical complexity, such as the need for advanced microfluidics training, and scalable infrastructure for high parallelization of perfusion systems. To overcome these complexity and biosafety limitations, we developed a novel, self-contained lung-on-chip platform. Our system operates using gravity-driven, rocking-based perfusion, which completely eliminates the need for external pumps and tubing. This integrated design drastically simplifies operation, enhances biosafety in infectious disease modeling, and makes advanced organ-on-chip technology accessible to any standard cell culture laboratory.

**Fig. 1:**
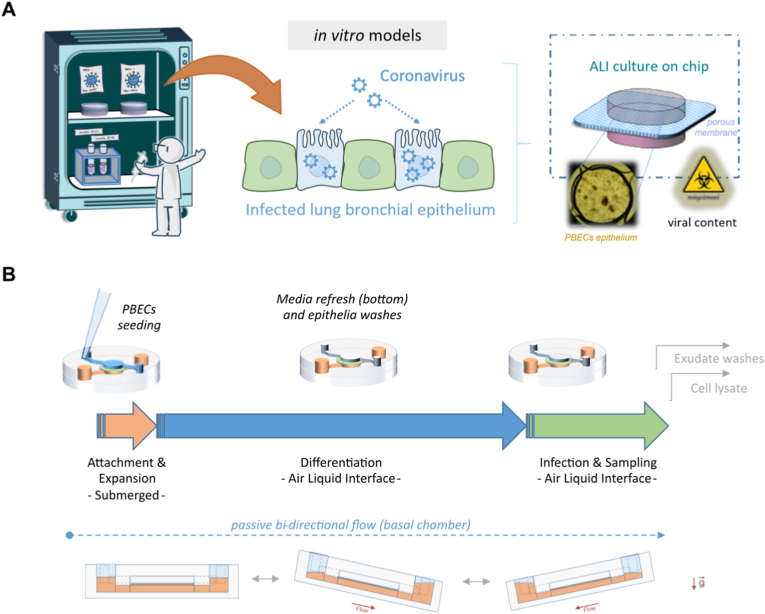
**Concept illustration on high-risk assays for testing viral infection *in vitro*. A)** Pathogens, like human coronaviruses (HCoVs) and SARS-CoV-2 variants, tested on epithelial *in vitro* models have to be handled carefully under high safety procedures and adequate infrastructure, risks extending to organs on chips typically requiring connection to external media sources. **B)** Summary of cell culture timeline for growth and differentiation of hPBECs on chip, including maturation at ALI, and viral infection and analysis.

In this study, we developed a novel enclosed LoC model, with a maximized cell culture area for approximately 28.27 mm^2^, easy to use under high containment, hPBECs were grown and differentiated into a mucociliary epithelial monolayer under ALI over 3–4 weeks and therefore exposed the mucociliary epithelium to viral infection. This enabled controlled infection with e HCoV-NL63 coronavirus, resulting in viral replication that followed the expected replication kinetics. Samples were easily retrieved for microscopy and gene expression analysis. We confirmed the upregulation of key antiviral gene transcripts from cell lysates. Using the physiological relevance of hPBECs in an easy-to-deploy device, we show that LoCs can be operated via standard micro-pipetting techniques and are ready for biosafety containment usage with minor deviation from routine procedures (Fig. 1A and B).

## 2 Materials and methods

### 2.1 Microfluidics manufacturing

#### 2.1.1 Design and rational of operation

**Fig. 2:**
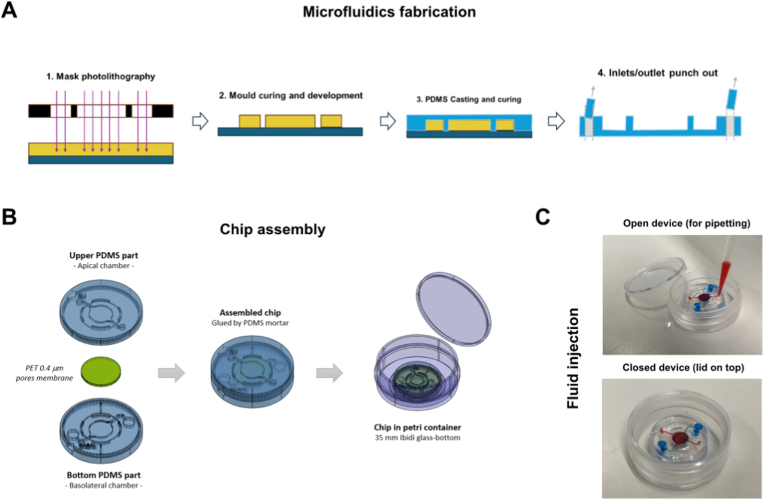
**Fabrication, assembly and test of the LoC devices. A)** Fabrication of the mold via photolithography. For this, the corresponding mask is placed onto the SU-8-coated silicon wafer to selectively crosslink the region of interest, after which the resin is baked and ultimately developed. Then, PDMS is casted on top of the mold and subsequently cured. After curing, the in- and outlets are created by punching. **B)** Assembly of the two casted PDMS halves. A PET membrane with 0.4 μm diameter pores is confined between the two PMDS halves using PDMS mortar with n-hexane. The chip is further bonded to the glass bottom of a Petri dish. **C)** Operational test of dual chamber devices, ie. apical (upper) and basolateral (bottom), for the independent injection and perfusion of 2 liquids (red, blue) in contiguous culture chambers, separated by the PET membrane.

The microfluidic chip is designed and manufactured as an enclosed system, compatible with culturing cells at biosafety levels three or higher. The fabrication of the LoC device involves a three-step process. First, photolithography is employed to create SU-8-silicon molds. Next, soft lithography is used to replicate the microfluidic structures into PDMS, and the assembly incorporates a porous membrane to separate the apical from the basolateral compartment (Fig. 2A). Finally, the assembled device is integrated into a Petri cell culture dish equipped with a tightly fitting lid closure, ensuring a secure seal, that also minimizes evaporation (Fig. 2B). The chip's top and bottom chambers are separated by a permeable culture membrane and equipped with independent inlets and outlets. The full microfluidic assembly is completed by encapsulation into a 35 mm diameter glass-bottom Petri dish (ibidi GmbH), sealed for sterility and ready for cell culture. The lower chamber is provided with larger-diameter inlet and outlet (3 mm; punched through the upper chip housing half to the bottom chamber) to act as extended medium reservoirs (Fig. 1B; Fig. 2C, in scale device). In contrast, the epithelial chamber is accessed by smaller-diameter inlet and outlet (1.2 mm; just on the upper side) to assure air permeability with minimal impact of access on unattended contamination.

#### 2.1.2 Gravity-driven flow numerical simulation

Computational simulation of velocity field and pressure was done by finite element modelling (FEM) using COMSOL Multiphysics ® 6.2 software. For this analysis, we chose the geometry restricted to the basolateral (bottom) compartment, given that during most of the culture duration the apical side will be exposed to air rather than to medium (liquid). The basolateral compartment comprised inlet, middle chamber, outlet and connecting channel between those. The medium flowing through the pores of the membrane is neglected as a major resistance largely prevents the fluid from passing through. The model simulations were defined using the Laminar Flow interface (governed by Navier-Stokes equations, Re < 1, neglected inertia, and incompressible flow) for computational fluid dynamics [44,45].

#### 2.1.3 Fabrication and assembly

The LoC device comprised a top and a bottom housing half from polydimethylsiloxane (PDMS) containing microfluidic structures, and a 10 μm thin ion track-etched porous membrane from polyester (polyethylene terephthalate, PET) containing pores with a diameter of 0.4 μm and a density of 6x10^6^ cm^−2^ (it4ip), which was sandwiched between the two housing halves. The housing halves were fabricated by casting PDMS onto a master mold produced from SU-8 photoresist (Kayaku Advanced Materials, USA) by UV photolithography on a 4-inch-diameter silicon wafer (Fig. 2A). In brief, an SU-8 layer of approximately 800 μm was coated in a single step on the wafer, underwent a two-step soft bake on a HP61A-2 programmable hot plate (Torrey Pines Scientific), for 30 min at 65 °C and 120 min at 95 °C, and then allowed to cool slowly to 25 °C. Through a high-resolution polyester film mask (Micro Lithography Services), the resist layer was exposed twice, each time for 15 s in a UV LED-based exposure-masking system (KLOÉ KUB-2; 365 nm; intensity/power: 25 mW cm^−2^), followed by a two-step post-exposure bake, for 10 min at 65 °C and 30 min at 95 °C, and then allowed to cool slowly to 25 °C. The (exposed) resist was developed in propylene glycol monomethyl ether acetate (PGMEA; Sigma-Aldrich) with gentle agitation, rinsed with PGMEA and in ultrapure isopropanol, and then blow-dried with a nitrogen gun. The PDMS prepolymer and curing agent (SYLGARD 184, Dow) were mixed in a 10:1 w/w ratio, cast on the mold, degassed in a vacuum desiccator, and then cured at 80 °C for 1 h. The resulting PDMS layer with microfluidic structures was peeled off and diced, and the inlet and outlet ports in the form of through-holes were punched into the later top housing half. The bonding of the housing halves and enclosure of the membrane (Fig. 2B) was done using a mortar from uncured PDMS precursor mixed with n-hexane at a 2:1 ratio. The mortar was dispensed onto the center of a plastic 12 cm Petri dish, where it was spin-coated at 1000 rpm for 30 s. The sides of the PDMS halves with the microfluidic structures were stamped onto the mortar coating, carefully avoiding a potential filling of the structures with the mortar. The housing halves were stacked onto each other with the PET membrane embedded in between at the center, using alignment marks. The sandwich was then allowed to settle for 30 min and baked in an oven for 2 h at 65 °C. Afterward, the PDMS sandwich chip was bonded into a 35 mm glass-bottom Petri dish, by means of O_2_ plasma, using 40 W for 45 s (Diener electronic Femto, before conformal contact, with alignment at the center) – also contributing to cleaning and sterilization. Prior to assembly, all components were handled in a horizontal laminar flow cabinet (HZ-2, Cruma). The microfluidic chips were then sterilized by exposure to germicidal UV-C light for 15 min.

#### 2.1.4 Device characterization

The SU-8-silicon molds were characterized by confocal laser-based optical profilometry (Keyence VK-X250K), using 10x magnification and large-area acquisition with 3D image stitching. Surfaces were leveled prior to quantification.

Using colored solutions of food dyes, the assembly of the LoC devices was validated for leakage-free gravity-based perfusion. To visualize the **l**iquid displacement, red and blue dyes were added to the basolateral (bottom) and apical (top) chambers, respectively. For this, small and large pipette tips press-fitted into the access ports were used as reservoirs for the LoCs’ top and the bottom compartments, respectively (tips are only illustrative or temporary, final assembly for cell culture has no tips attached or additional volume off chip). By tilting the chip, the solutions move towards the lowered side, perfusing the microfluidic compartments.

### 2.2 Cell culture

#### 2.2.1 Patient material

Lung tissue samples were collected from lung lobe resections performed at the Erasmus Medical Center (Rotterdam, the Netherlands). All lung tissue collected followed the “Human Tissue and Medical Research: Code of conduct for responsible use (2011, https://www.coreon.org/wp-content/uploads/2020/04/coreon-code-of-conduct-english.pdf)” guidelines. Informed consent was obtained from patients and approved by the Medical Ethical Committee (METC nr. MEC-2012-512). All donor samples were from males between 50 and 65 years old.

#### 2.2.2 Isolation of primary cells

hPBECs were isolated from resected tumor-free bronchial tissue as described previously [46]. In short, hPBECs were isolated from a human bronchus ring, which was incubated in 0.18 % protease type XIV (Sigma, cat. no. P5147-1G) in Dulbecco's phosphate-buffered saline (DPBS; Sigma-Aldrich) at 37 °C for 2 h. Afterward, the bronchus ring was scraped using a surgical knife to release the cells into a Petri dish. The loose tissue and cells were collected in complete keratinocyte serum-free medium (KSFM; Supplementary Table 1) and plated on a pre-coated 6-well plate. Cells were trypsinized after reaching confluence and frozen for further use.

#### 2.2.3 Seeding on chip and differentiation at ALI

The upper, apical compartment of the chip was filled with 20 μl of hPBEC coating solution (Supplementary Table 1) and incubated at 37 °C for 2 h. The solution in the apical compartment was aspirated and the compartment washed once with DPBS. A total of 5-8x10^4^ hPBECs in 20 μl complete bronchial epithelial cell growth medium (BEGM; Supplementary Table 1) was seeded per chip (apical side). The lower, basolateral compartment was filled with 100 μl of complete BEGM. All chips were placed on a rocker with a cycle speed of 2 cycles per min. After 16–24 h, the medium was refreshed. Three days later, the medium in the basolateral compartment was changed to complete BEGM + EC23 50 nM (Tocris, cat no. 4011) and the medium in the apical compartment was removed to create an ALI. Cells were allowed to differentiate for 2.5–3 weeks during which the medium was refreshed 3x per week.

#### 2.2.4 Epithelium infection by HCoV-NL63 on-chip

The HCoV-NL63 virus stock was generated by inoculating cells of the rhesus monkey kidney epithelial cell line LLC-MK2 with the virus and incubating them at 33 °C [47]. Viral particles were applied to the apical side of a differentiated mucociliary epithelium. Therefore, 20 μl of viral stocks (approximately 4.5x10^8^ viral RNA copies) were added to the apical compartment of the chips and incubated for 2 h at 33 °C. This infection was repeated twice, subsequently the apical side was washed 3x with 100 μl DPBS + Ca^2+^ + Mg^2+^. Dulbecco's Modified Eagle's Medium (DMEM; STEMCELL Technologies) was used as mock infection control. After 1 h at 33 °C, an initial 100 μl was taken and mixed with an equal amount of RLT buffer (QIAGEN, cat. no. 74106) as a reference point for infection. Apical washes were collected at 48 h, 96 h and 7 days, and mixed with RLT buffer as well. Remdesivir 1 μM (MCE, cat. no. HY-104077) was added to assess mitigation of viral replication.

#### 2.2.5 Immunocytochemistry

The membranes were recovered from the chips with a razor blade and fixed in 4 % (w/v) paraformaldehyde (PFA; Sigma-Aldrich, cat. no. 441244) at room temperature (RT) for 15 min. The membranes were processed as previously reported [39,48]. Membranes were washed 3x with PBS and subsequently blocked for 1 h at RT in 5 % normal donkey serum (Sigma Aldrich, cat. no. S30-100 ML), 1 % bovine serum albumin (BSA; Roche, cat. no. 10735086001), and 0.3 % Triton X-100 (Sigma, cat. no. T8787) in PBS. Primary antibodies (Supplementary Table 2) were diluted in blocking buffer and incubated over night at 4 °C. The next day, the membranes were washed 3x with 0.03 % Triton X-100 in PBS for 10 min. Secondary antibodies (Supplementary Table 2) were diluted 1:500 in blocking buffer together with 4′,6-diamidino-2-phenylindole (DAPI) solution (stock: 1 mg/ml) 1:2000 (BD Biosciences, cat. no. 564907) and incubated in the dark for 2 h at RT. Next, membranes were washed 3x with 0.03 % Triton X-100 in PBS for 10 min. The membranes were transferred into PBS and mounted onto a microscope slide (Epredia, cat. no. J1800AMNZ) with a coverslip (Menzel-Gläser; 24 × 60 mm^2^) using mounting reagent consisting of 2.4 % (w/v) Mowiol (Sigma-Aldrich, cat. no. 81381), 12 % (w/v) 0.2 M Tris pH 8.5 (Sigma-Aldrich, cat. no. T6066) and 4,75 % (v/v) glycerol (Honeywell, cat. no. 49770-1L) in ddH_2_O. Images were made using a confocal fluorescence microscope (Leica SP5).

#### 2.2.6 Gene expression analysis

Cells were lysed on the membrane in the chip by injecting the chips with 150 μl of RLT buffer of the QIAGEN mini RNeasy kit (Qiagen, cat. no. 74136) through a P200 filter tip in the inlet port towards another P200 filter tip in the outlet port and washed with an additional 150 μl RLT buffer. Both lysates of the cells were collected into one sampling tube. Both the cell lysis RNA and viral exudate washes were further processed as stated in the protocol of the QAIGEN mini RNeasy kit (Qiagen, cat. no. 74136). The purified RNA was used to prepare cDNA using the iScript cDNA synthesis kit from Bio-rad (Bio-Rad, cat. no. 1708891). RT-qPCR was performed with gene-specific primers (Supplementary Table 3) combined with SYBR Green Universal Master Mix (Applied Biosystems, cat. no. 4364346) in 96-well plates (Roche, cat. no. 04729692001). The RT-qPCR program was set to 95 °C for 10 min, followed by 45 cycles of 95 °C for 10 s, 58 °C for 30 s and 72 °C for 30 s. Data was normalized with glyceraldehyde 3-phosphate dehydrogenase (GAPDH) as an internal standard and further analyzed using Excel. Fold-increase normalization with the mock infection was used in comparison to the viral titer. The data was displayed using GraphPad Prism 10.2.3.

#### 2.2.7 Quantification of HCoV-NL63 viral copies

The HCoV-NL63 viral copies were calculated using the following equation: copy number (molecules/μl) = [concentration (ng/μl) × 6.022 × 10^23^ (molecules/mol)]/[length of amplicon × 660 (g/mol) × 10^9^ (ng/g)] this led to a previous generated formula ‘y = −0.3345x+13.137’ (y equals the log_10_ of viral RNA copy number, derived from a standard curve based on plasmid DNA standards; x equals the qPCR Ct value) [47,49]. In total, 2 μl of cDNA from the lysed cells was used in the RT-qPCR. This multiplied with the total produced cDNA per chip results in the number of viral copies per lysate. To account for minor variations in RNA yield across chips and donors, viral copy number was normalized to the amount of RNA input used for cDNA synthesis.

#### 2.2.8 Statistics

All statistical analyses were performed using GraphPad Prism version 10.2.3. Data are presented as mean ± standard deviation (SD). Differences between the two groups, mock-infected chips and infected chips, were assessed using an unpaired Student's t-test or, for comparisons involving three or more groups, one-way ANOVA was performed. For both tests, data were assumed to be normally distributed. Benjamini-Hochberg procedure was applied to control the false-discovery rate and the p-values were adjusted accordingly. The study included a total of three male donors (N = 3) between 50 and 65 years old, with each experimental condition having 3 or more technical replicates (n > 3).

## 3 Results

### 3.1 Chip manufacturing and characterization

**Fig. 3:**
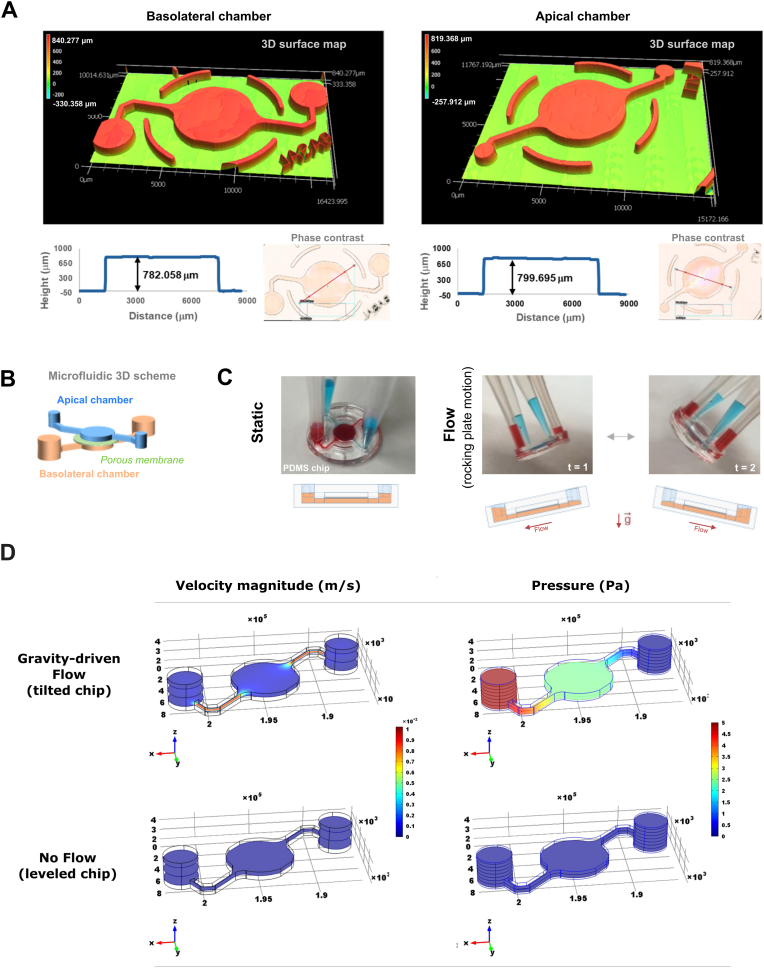
**Characterization of molds for microfluidic components and geometry testing for media flow. A)** Profilometry data of the cast of the (bottom side chamber) basolateral and (upper side chamber) apical compartment in PDMS. **B)** Concept schematic of microfluidic network with both compartments separated by porous membrane. **C)** Fluidic testing of the chip. Red and blue dyes are loaded into the chambers using pipette tips. By tilting the cast, the fluid flows to the other chamber. D) Finite Element Modeling (FEM) of Gravity-driven Flow for the basolateral chamber of the microfluidic chip, assuming an initial volume imbalance of 1.77 μL (equivalent to 1.77 mg of water) at the inlet, and that this volume is displaced in 10 s, as it moves toward the outlet due to gravity. The results show relative values, as real-world flow conditions depend on the rocking platform's tilt angle, angular speed (duration of cycles) and the total volume of the fluid circuit. The model illustrates two conditions. (Flow) Shows the velocity field under “gravity-driven flow”, with the highest velocities in the narrow channels and more moderate flow in the basolateral chamber. (Static) Represents the "no flow" condition, which is assumed when the chip is kept perfectly horizontal with a balanced liquid volume at both the inlet and outlet (no velocity of fluid). On the tilted chip, the pressure is highest at the inlet due to the force exerted by the accumulated liquid mass. This creates a pressure gradient that drives the flow through the channel, causing the pressure to decrease gradually towards the outlet. For visualization, the velocity field (m/s) and pressure (Pa) are shown on 3 and 5 horizontal planes, respectively, with one plane always positioned at the level of the main chamber.

Laser optical profilometry was used to assess the quality of the fabricated molds for the microfluidic components (Fig. 3A). Measurements showed a well-resolved 3D shape with straight walls and distinct features. The average height of the microfluidic structures was approximately 800 μm (Fig. 3A). The device features an apical (top) chamber and a basolateral (bottom) chamber, separated by a porous membrane. The basolateral chamber has wide access inlets with 4 mm diameter, which can be punched through and serve as reservoirs for medium delivery with a micropipette, accommodating up to 40 μl on each side for feeding the cell culture from the lower compartment (Fig. 3B). The apical chamber has smaller inlets with 1.2 mm diameter, designed primarily for seeding the cells of the epithelial layer, and deliberately having narrowed access ports to prevent dehydration and limit access to external particulates. This chamber supports 10.13039/501100010665ALI culture 24 h after seeding, facilitating a second culture phase required for cell differentiation. This feature is crucial for achieving the physiological relevance of the cultured tissue.

Medium flow was tested by adding red dye to the basolateral chamber and blue dye to the apical chamber and tilting the chip to visualize liquid displacement (Fig. 3C). Flow was observed to be higher in the basolateral compartment, a result attributed to its lower hydrodynamic resistance and the greater gravitational pressure exerted by the larger fluid volume. Importantly, no cross-chamber leakage of the dyes was detected.

The symmetrical design of the chip is critical, as it allows a rocking platform to generate passive, gravity-driven flow that is both bidirectional (from the elevated side to the lower side) and produces an equivalent flow profile in each direction. In the device, the flow velocity is determined by the volume of the culture medium, the tilt angle, and the frequency of the rocking motion. To characterize these fluid dynamics, we performed finite-element modeling (FEM). As presented in Fig. 3D, the simulation shows the velocity fields and pressure maps of medium under two distinct states: dynamic (during gravity-driven rocking motion) and static (when the chip is stable, horizontal position).

### 3.2 On-chip maturation of a bronchial epithelium at ALI

**Fig. 4:**
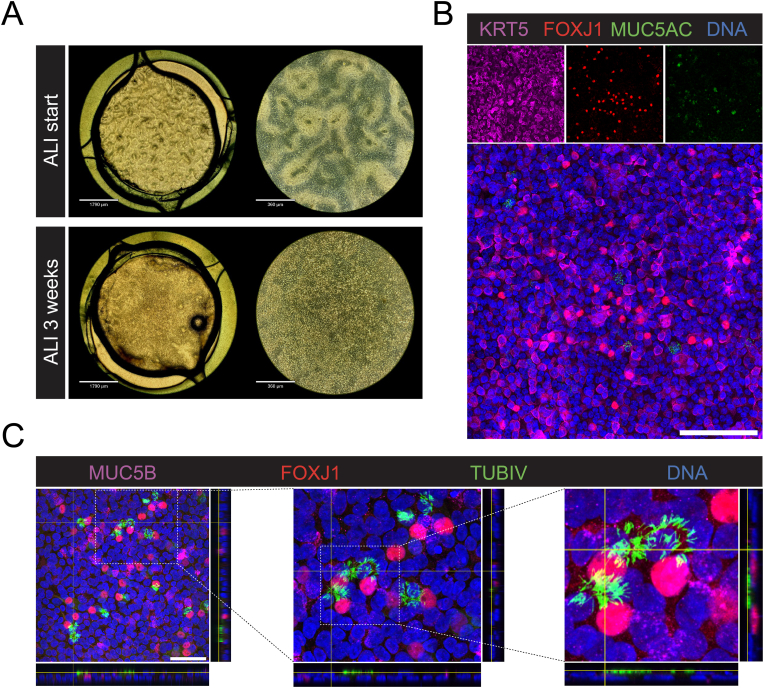
**Characterization of the hPBEC layer on the concealed LoC. A)** Brightfield microscopy images captured at the initiation of the ALI culture and after three weeks of culturing, prior to infection studies. The cells had a distinct cobblestone epithelium layer without irregularities over a period of 3 weeks. Scalebar of left images is 1790 μm and on the right 360 μm. **B)** Confocal fluorescence microscopy images of the hPBECs in the concealed lung-on-chip device after 3 weeks of ALI culture. The cells are stained for KRT5 (magenta), FOXJ1 (red), MUC5AC (green) and DNA (blue). The top three smaller images are from individual channels, the larger bottom one is the merged channel including the DNA staining. Scale bar represents 200 μm. **C)** Confocal fluorescence microscopy images of MUC5B (magenta), FOXJ1 (red), TUBIV (green) and DNA (blue). XZ and YZ cross-sectional planes are depicted on the sides of the images. Scale bar represents 100 μm and applies for all images in the subfigure.

We first tested different membrane types to evaluate the most optimal supporting material for cell adhesion and confluent epithelium integrity up to 1 week (Supplementary Fig. 1). Seeding the cells in chips containing different types of ion track-etched PC and PET membranes resulted in variations in the levels of cell adherence and differentiation. Long-term hPBEC cultures were best supported by a PET membrane with 0.4 μm pore diameter and a pore density of 6x10^6^ cm^−2^ as determined by the differentiation of the epithelial cells, and the absence of patches without cells resulting from detachment of the cell monolayer. A combination of collagen I, BSA, and fibronectin was used as coating for cell adherence and rapid cobblestone layer formation (Fig. 4A), in line with our prior studies [29,39].

One of the key characteristics of hPBECs is their ability to differentiate into a mucociliary layer when grown at ALI, effectively mimicking the airway epithelia *in vitro*. To achieve visible cell layer differentiation typically takes over two weeks. Following the successful seeding of the chips, the cultures were maintained submerged for 3–4 days before removing the medium in the apical compartment. The ALI condition could be maintained for over 3 weeks (Fig. 4A). After fixing the chips, immunofluorescence staining with specific antibodies showed a confluent KRT5^POS^ basal layer with the presence of FOXJ1^POS^ and TUBIV^POS^ ciliated cells and MUC5AC^POS^ and MUC5B^POS^ secretory cells (Fig. 4B and C). The 3D reconstruction from a z-stack of confocal microscopy images showed the presence of TUBIV^POS^ cilia at the apical side of the epithelial cells (Fig. 4C).

The properties of the selected membrane, particularly its material chemistry and permeability, proved critical for supporting the hydration, integrity, and differentiation of the epithelial layer, as demonstrated in our optimization study **(**Supplementary Fig. 1). Nevertheless, prolonged culturing required continuous monitoring because irregularities such as air bubble formation would occur, which in case of drying underlying water content would also impede water hydration of culture through membrane. Hence, culture maintenance by regularly refreshing medium and performing apical washes was necessary to minimize the impact of such irregularities on the maturation of the epithelial cells. Similarly, eventual condensates on upper chamber might break the ALI condition, for which continuous monitoring and clearing was relevant towards mucociliary maturation.

### 3.3 Modelling coronavirus NL63 infection and antiviral treatment in LoC

**Fig. 5:**
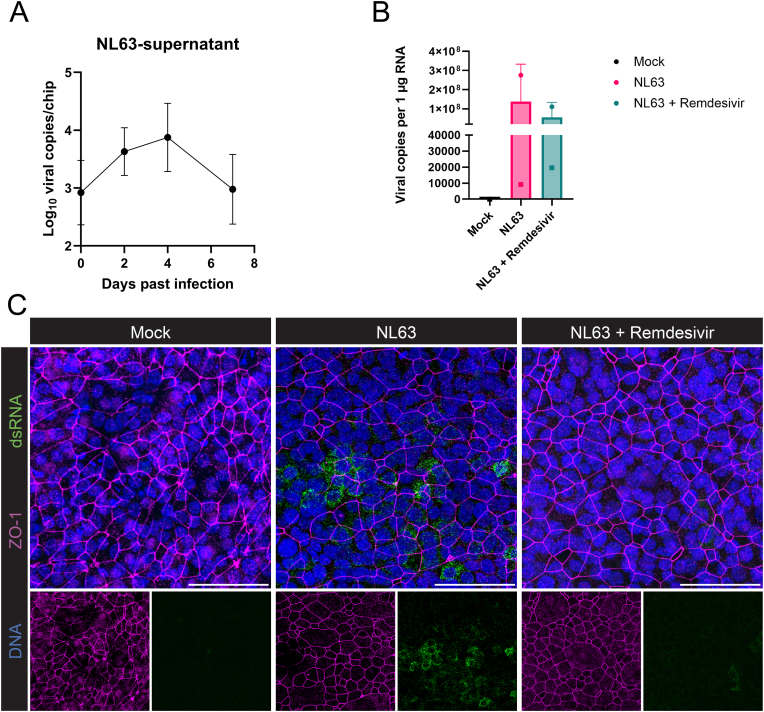
**Infection profile of the HCoV-NL63 virus strain. A)** Viral copies found in the apical lysate washes of infected chips from one donor over time (n = 6), data presented as mean with SD error bars. **B)** Viral copies per 1 μg of RNA in the cell lysates 7 days after infection (N = 2, n ≥ 3), data presented as mean of one donor (corresponding symbols in different conditions) with SD error bars. Statistical significance was tested between conditions via one-way ANOVA but did not reveal any significance. **C)** Immunofluorescence staining of the infected chips. For each condition, the top image shows a merged view of the two lower images. The chips were harvested on day 4 after infection and stained for ZO1 (magenta), dsRNA (green) and DNA (blue). Scale bars represent 50 μm.

We next tested the feasibility to use this LoC to model respiratory virus infections. We utilized the relatively harmless HCoV-NL63 virus strain as a representative model for coronaviruses, because it shares a common host cell virus receptor (ACE2) with SARS-like coronaviruses The epithelium was infected with HCoV-NL63 at least 2.5 weeks after initiation of the ALI. Apical washes were performed and collected post infection to measure the virus secreted by the epithelial cells. The apical washes were studied over a 7-day period by performing a virus-specific qPCR, and an increase in viral copies was observed during the first 4–5 days after which the titer declined (Fig. 5A). Next, we evaluated whether the hPBECs had differentiated into a functional mucociliary epithelium to form a tight barrier at day 5 post infection, at the height of the virus infection. Indeed, staining the cultures with ZO-1 displayed a cobblestone pattern indicative of a dense array of intercellular junctions (Fig. 4, Fig. 5C). The total viral RNA copies per 1 μg of cDNA made from the cell lysates of day 5 and 7 of on-chip culture after infection was quantified (Fig. 5B), showing the differences between donors. Data from both donors were pooled for statistical analysis between conditions, but no statistically significant difference were detected. Remdesivir showed its antiviral properties by inducing a trend in the decrease of the viral copies per area of hPBEC layer in the chip (Fig. 5B). This infection efficiency was demonstrated by immunolabeling for virus-replicating intermediate double-stranded RNA (dsRNA). As expected, dsRNA was found in the HCoV-NL63 infected chips and not in the mock-infected chips (Fig. 5C). In contrast, infection of samples treated with remdesivir, a proven antiviral drug inhibiting the viral RNA polymerase [50], showed fewer infected cells compared to the non-treated HCoV-NL63-infected on-chip cultures.

### 3.4 Cellular mechanism of response to viral infection by HCoV-NL63 are activated with intrinsic donor variability

Interferon signaling, as the first line of innate defense against many viral infections, can activate interferon-stimulated-gene (ISG) transcription and establish an antiviral state. To specifically map whether HCoV-NL63 infection can trigger such host antiviral response in the LoC model, we monitor the expression of a panel of ISGs, such as *ISG15, MX1* and *IFIT* [17,51,52].

**Fig. 6:**
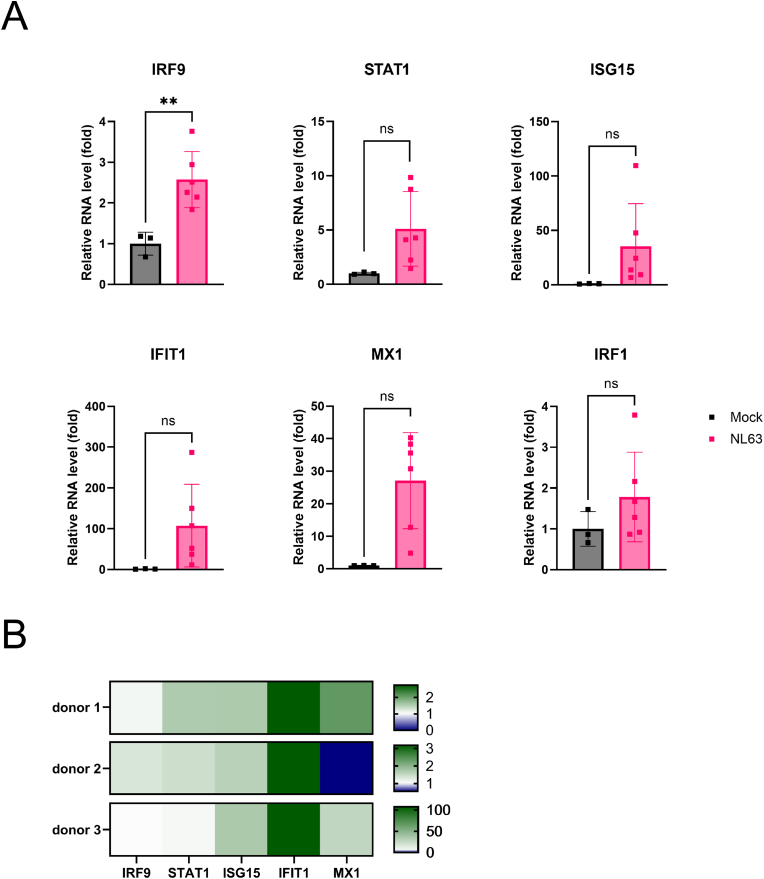
**Cell viral machinery dysregulation and response to viral infections.** RNA is harvested from the cell lysates from day 4 or 7 after infection for RT-qPCR. **A)** Viral gene panel measured via RT-qPCR that is upregulated during the infection of HCoV-NL63. The genes are normalized to GAPDH and subsequently normalized to the control for the fold change (n ≥ 3), day 4 after infection. Statistical significance was assessed using an unpaired *t*-test. False-discovery-rate was applied to the p-values, adjusted p values are shown. ∗ = p < 0.05, ∗∗ = p < 0.01. **B)** Heatmap showing the fold change normalized to the mock infected chips of the viral genes in different donors (n ≥ 3) with 0 set to blue, 1 to white and the top expression of that donor to green. RNA was harvested after infection at day 4 for donor 1 and 2 and on day 7 for donor 3.

An initial screening for pathway activation on HCoV-NL63-infected mature epithelia cultured at ALI revealed significant activation of immune response pathways to viral infection (Fig. 6A). All selected genes show a steep increase in expression, suggesting that the machinery dedicated to remove the viral infection was activated upon infection. Especially, *IRF9*, involved in the activation of antiviral genes [53], is significantly upregulated. Moreover, *MX1*, involved in blocking the viral transcription cycle [54], shows a steep increase. Further analysis of additional donors for the same pathways showed considerable variability in gene expression and heterogeneity in the principal mechanisms of response. This donor variation is visually summarized in a heat map, illustrating individualized donor gene expression levels for the pathways analyzed (Fig. 6B). We found several genes to be similarly upregulated in all donors, i.e. *IFIT1*, *ISG15* and to a lesser extent *IRF9*. In contrast, *STAT1* and *MX1* show high variability in activation levels across donors.

## 4 Discussion

Respiratory infections remain a major contributor to healthcare expenditure, requiring extensive medical personnel involvement and infrastructure resources. There is an urgent need for more advanced human *in vitro* models to accurately study respiratory tract infections and therefore improve treatments. In this study, we introduce a novel device designed to enhance biosafety containment, a crucial aspect when handling infectious pathogens or particles in microphysiological systems, particularly LoC devices used for respiratory virus studies. Operating in a tubeless configuration, the device has inherent limitations regarding fluid dynamics but effectively mitigates the risks associated with external access to the high-risk system handling viral vectors. Despite progress in LoC technologies, most systems still depend on external tubing, require specialized training, and face scalability issues [38,43]. While advanced chips support multiple cell types for enhanced physiological relevance, their complexity often hinders reproducibility and handling - especially in infectious disease studies. Active pumping further increases contamination risks due to leakage or delamination. A critical next step for the field is the systematic comparison between different lung-on-chip platforms and traditional static ALI cultures, such as porous membrane inserts. Such validation studies are essential for the standardization and qualification of these platforms as New Approach Methodologies (NAMs) [[55], [56], [57]]. Ultimately, robust and well-characterized NAMs hold the potential to reduce the reliance on animal models in non-clinical testing, offering a more human-relevant approach to assessing the quality, efficacy, and safety of new pharmaceutical products.

We developed a self-contained LoC platform that supports differentiated primary lung cell cultures without external tubing or pumps. It enables HCoV-NL63 infection studies in bronchial epithelial cells and features design elements - such as rounded microfluidic corners, a circular chamber to reduce bubble formation, and elevated ALI compartments to prevent condensation - to prevent water or mucus buildup that could impair mucociliary differentiation. Encased in a gas-permeable, sealable 35 mm Petri dish, the device enhances biosafety and usability. It is compatible with standard micropipettes (P20/P200) for direct liquid injection, requires minimal training, and eliminates the need for external reservoirs, making it suitable for high-containment workflows. Such simple approach democratizes organ-on-chip technology, that can be used in almost any standard biology lab, not just a specialized engineering or microfluidics lab. Operating under pump-less conditions, the system eliminates the need for external tubing for viral particle delivery and continuous feeding pressurization. The gravity-driven flow within the microfluidic chip facilitated by the rocking motion of the Petri dish, provided sufficient medium flow to maintain the cultures for extended periods. This supported the epithelial layer's 10.13039/501100010665ALI differentiation over the two-week culture period until infection and continued to do so during the post-infection period, while maintaining viable epithelial cells and sustained viral infections within the system. Nevertheless, while our gravity-driven flow is highly effective for maintaining the ALI culture of PBECs, it does not provide the precise, programmable flow control offered by advanced pump systems. This level of control may be a key requirement for certain applications, such as quantitative shear stress studies or pharmacokinetic modeling [58].

While the LoC model offers a promising platform for studying airway tract infections and their disease mechanisms, further optimization is still required. One key challenge relates to the prevention of water condensation in the chips, which seemed to be also influenced by variations in the manufacturing of the chips. Some chips performed excellently in this concern, while others needed to be aspirated during regular medium exchange to keep an ALI. As mentioned, this might have limited mucociliary differentiation. Supplementary Table 4 provides a troubleshooting guide listing common problems encountered during device handling and their corresponding solutions. Additionally, the manufacturing and operation of the chips can be upscaled towards medium throughput, as shown in this paper, while still relying on casting methods. However, upscaling production for broader application would require transitioning to injection-molded components. While this would dramatically increase fabrication output and enable more extensive testing, a key challenge will be ensuring that the new materials/configuration maintains adequate culture oxygenation. Another key aspect of organ-on-chip upscaling is the inherent operational complexity. High-throughput platforms present significant logistical challenges, as managing numerous chips in parallel for tasks like microscopy and frequent media exchanges is demanding and often requires automation. Notably, Fisher et al. [38] presented a high-throughput airway-on-chip platform for modeling SARS-CoV-2 infection using a 96-well plate format driven by a custom-made 192-pump array. The massive parallelization of such systems is invaluable for efficiently screening drug candidates, exploring a wide range of experimental conditions, and addressing donor variability by increasing the number of samples per run. Our approach, however, was specifically engineered to create a simple, pump-less, mid-throughput, and off-the-shelf device whose key advantage is its accessibility.

The differentiation of the hPBECs in the chip is an essential step in replicating the airway and modeling the infection, given the need for mucociliated epithelia for efficient viral infection. To optimize the LoC, we decided to study the dynamics and effects of viral infections with a less virulent coronavirus with the same mode of entry as SARS-CoV-2. Human coronaviruses, including SARS-CoV-2 and the common cold coronaviruses HCoV-NL63, HCoV -OC43, and HCoV -HKU1, consistently target ciliated cells [[59], [60], [61], [62], [63]]. The mechanisms by which these viruses affect the airway can be quite heterogeneous [9,16,25,64]. Interestingly, SARS-CoV-2 infects both ciliated and secretory cells in human airway epithelium (HAE) cultures, replicating with high efficiency [61]. In contrast, HCoV-NL63 is only detected in ciliated cells and replicates with lower titers [63]. Notably, both viruses share the same receptor, angiotensin-converting enzyme 2 (ACE2), and transmembrane protease, serine 2 (TMPRSS2), which are expressed in both HAE and lung parenchyma, typically more abundantly on the apical side of polarized airway epithelia [12,14].

In our LoC model, we observed differentiation towards a mucociliary epithelium over the course of 3–4 weeks after inducing an ALI. Secretory cells were depicted via MUC5AC and MUC5B, while ciliated precursor and ciliated cells were visualized using FOXJ1 and TUBIV, respectively. Indeed, the successful differentiation of the airway epithelium on the chip provides an essential step for investigating the pathogenesis of respiratory infections. Equivalent level of maturity was found in comparison to other primary airway cultures in culture inserts at the ALI (for ciliated and secretory cells) [65]. These findings validate the capacity of the LoC platform to support robust epithelial differentiation, a critical step in modeling respiratory infections and their pathogenesis *in vitro*. The system's ability to replicate aspects of the functional complexity of the airway epithelium highlights its potential to study host-pathogen interactions.

The functionality of our airway epithelial model was validated by infecting differentiated hPBECs with the HCoV-NL63 virus. We monitored several aspects of the infectious process, such as viral shedding. Notably, the collection of the apical washes on the chip revealed an increase in viral RNA levels until day 4, decreasing thereafter. The decrease of dsRNA in the infected epithelia when treated with the inhibitor remdesivir compares to similar studies, e.g., in airway organoid cultures [47] and other on-chip cultures [38,66,67]. Additionally, the epithelial layer was kept intact as shown by the tight junction marker ZO1. In SARS-CoV-2 and influenza studies, it was found that 24h post infection the permeability of the barrier would increase [68,69]. A similar study with HCoV-NL63 viral infection showed a direct impact on the decrease of the transepithelial electrical resistance (TEER) at the apical side 96 h post infection [70]. However, no noticeable barrier degradation was found in the ZO1 patterning between any of the different conditions 96 h post infection. The current chip design does not accommodate direct TEER measurements. Future versions could incorporate embedded electrodes - such as gold (Au), platinum (Pt), or transparent electrodes from indium tin oxide (ITO) - to enable on-chip measurements while maintaining the field of view for microscopy, which would allow for the continuous monitoring of barrier function and provide a more comprehensive characterization of the cellular model. Alternatively, FITC-dextran permeability assays could be performed to provide a quantitative measurement of barrier degradation post-infection.

Moreover, we investigated the cellular response mechanisms triggered by the HCoV-NL63 viral infection. In response to viral infection of a host cell, the immune system activates a multi-layered defense mechanism to detect and eliminate the virus [9,71]. Mechanisms like pattern recognition, with receptors for dsDNA, which activate type 1 interferons (IFN-α and IFN-β), lead to the activation of the JAK-STAT signaling pathway, resulting in the activation of ISGs [[72], [73], [74]]. This chain represents the innate immune response acting as the first line of defense for viral clearing. Indeed, our data shows different upregulations of immune pathways in line with existing literature. We monitored the viral entry of the HCoV-NL63 virus via *IRF1* for pro-inflammatory genes and *STAT1* and *IRF9* for key reference ISGs [17]. Here, it activates multiple genes like *ISG15*, *MX1* and *IFIT*. *ISG15* encodes for a protein that interacts with viral proteins, ensuring the loss of function [51]. The activation of the interferon response was further validated by the upregulation of *MX1* and *IFIT1,* both have shown in prior virology studies to restrict viral replication, thereby reinforcing the relevance of the model to house viral infections [52,64,75]. These observations support the physiological relevance of the LoC model and its utility to recapitulate host-virus interactions during respiratory infections of HCoV-NL63. While this exploratory study focused on a single viral strain, the versatility of our platform allows for future investigations into other viruses. This could enable systematic comparisons of different viral strains, which are known to exhibit diverse modes of action and host-virus interactions [76,77] Furthermore, the adaptability of the model places it in a strong position for future integration with other cell types that benefit from utilizing an enclosed format, enabling more comprehensive studies of multi-organ infection.

Donor variations were found to play a part in the discrepancies in activation of the different antiviral pathways. For each antiviral pathway gene, cells from different donors exhibited different expression levels. During the COVID-19 outbreak, considerable differences were noticed between the responses of patients to viral infections from SARS-CoV-2 [78]. While this variance in susceptibility was found in patients, *in vitro* studies also displayed noticeable differences between donor activity after SARS-CoV-2 infections [37,79]. Moreover, this donor reactivity exists outside of viral infections. For example, an *in vitro* study found that different hPBEC donors showed variation in stress response to cigarette smoke [37]. This variance may have resulted in the reduction, though not significant, of the viral titer in the cell lysates from cultures conditioned with remdesivir [71].

## 5 Conclusion

In summary, the LoC developed in our study holds significant promise for advancing our understanding of viral infections, featuring unique deployability in any cell biology and virology laboratory. The LoC offers significant advantages in terms of ease of use, as it does not require complex equipment or external tubing. This also enhances the safety needed for working with viral agents. Using hPBECs, we demonstrate differentiation into a mucociliary cell layer that can be infected by the HCoV-NL63 coronavirus. The viral infection activates immunity genes in the bronchial epithelial cells. With ongoing improvements in microfluidic technology and model refinement, the proposed LoC platform further emerges as a tool for viral infection research and potential therapeutic developments, namely antiviral testing.

## CRediT authorship contribution statement

**David Barata:** Writing – review & editing, Writing – original draft, Visualization, Validation, Methodology, Investigation, Formal analysis. **Sem Koornneef:** Writing – review & editing, Writing – original draft, Visualization, Validation, Methodology, Investigation, Formal analysis. **Francesca Giacomini:** Writing – review & editing, Methodology, Investigation. **Zeinab Niloofar Tahmasebi Birgani:** Writing – review & editing, Supervision, Methodology. **Jiangrong Zhou:** Writing – review & editing, Validation, Methodology. **Pengfei Li:** Writing – review & editing, Methodology, Investigation. **Robbert J. Rottier:** Writing – review & editing, Validation, Supervision, Methodology, Funding acquisition, Conceptualization. **Roman K. Truckenmüller:** Writing – review & editing, Validation, Supervision, Methodology, Funding acquisition, Conceptualization.

## Declaration of competing interest

The authors declare that they have no known competing financial interests or personal relationships that could have appeared to influence the work reported in this article.

## Data Availability

The data that support the findings of this study are available from the corresponding author upon reasonable request.
