# Supplementary material for: Simple and high-containment lung-on-chip model for studying respiratory viral infections using human primary lung cells
Source: Mater Today Bio. 2025 Sep 15;35:102316. doi: 10.1016/j.mtbio.2025.102316 (PMC12494930; doi:10.1016/j.mtbio.2025.102316)
Supplement: Multimedia component 1 [file mmc1.docx]

**Supplementary Information**

for

**Simple and high-containment lung on chip model for studying respiratory viral infections using human primary lung cells**

**Supplementary Figure 1**


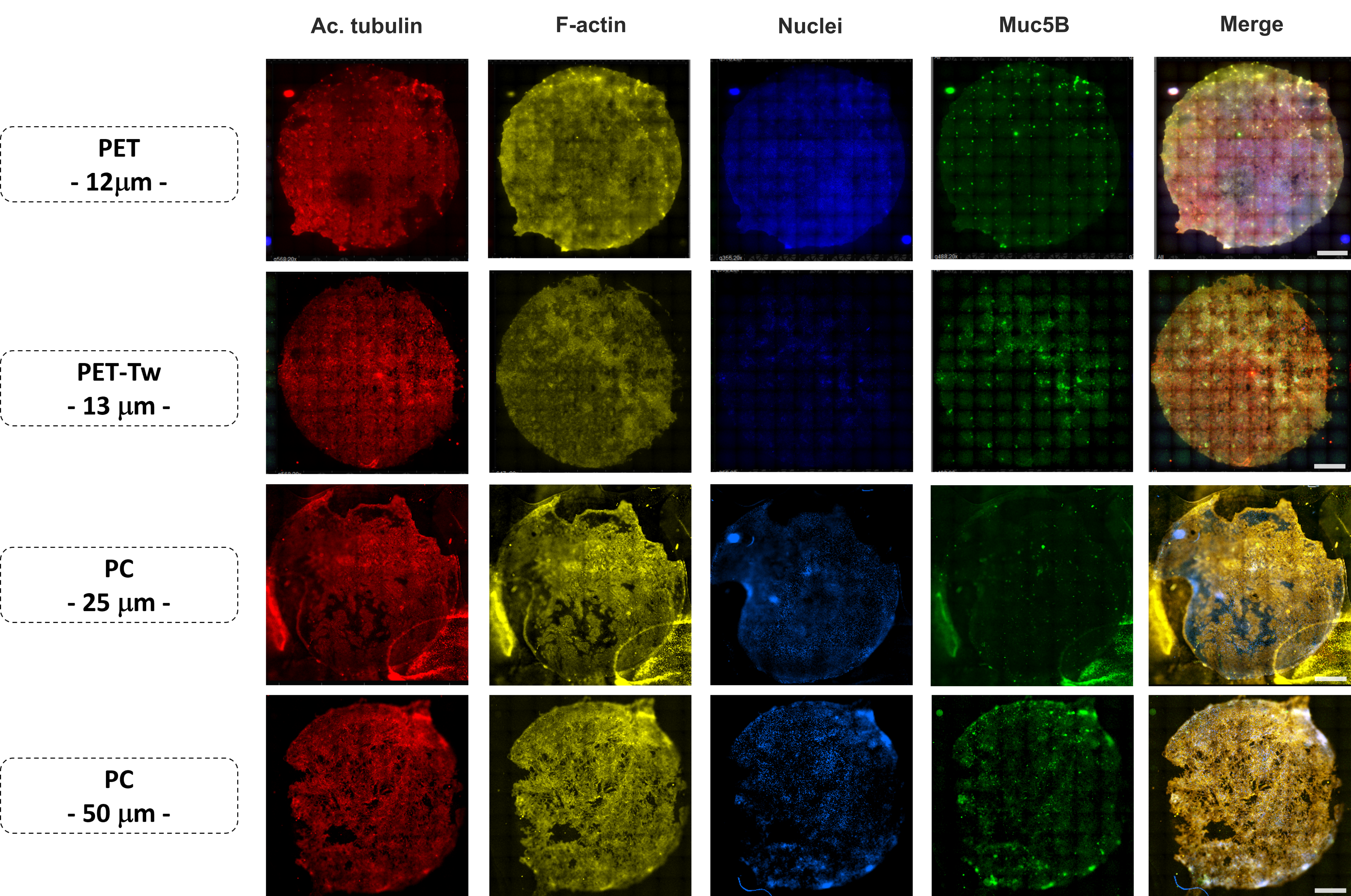


**Supplementary Figure 1:** Stitched fluorescence microscopy images of human primary bronchial epithelial cells (hPBECs) layers on different membrane types after 1 week of on-chip culture at ALI for the assessment of full area cell coverage (blue, nuclei, and yellow, F-actin for cytoskeleton) and biomarker expression for epithelial cells (red, acetylated tubulin) and mucinous cells (green, Muc5B). Five replicates were used for each condition. In both polycarbonate (PC) membrane chips, hPBECs only displayed 20% of viable chips (cell coverage >50%) after 7 days of culturing. The diameter of cell culture area was 6.0 mm. For each membrane type, the images represent the best performing samples (still showing minor empty patches, assumedly also due to manipulation of samples). Polyethylene terephthalate (PET) and PC membranes are track-etched membranes with transversal pores (pore diameter of 0.4 μm and a density of 6x10^6^ cm^-2^), and are cut out from larger sheets. PET-Tw are culture insert membrane (cut out from a Corning Transwell 3460 insert). Scale bars = 1 mm.

**Supplementary Table 1: Cell reagents used for culturing hPBECs**

| **Name** | **Component** | **Final concentration** | **Supplier (cat. No.)** |
| --- | --- | --- | --- |
| Coating | PureCol (PC) 3mg/mL | 30 µg/mL | Advanced Biomatrix (5005) |
|  | Human Fibronectin (FN) | 10 µg/mL | EDM Millipore (FC010) |
|  | Bovine Serum Albumin (BSA) | 10 µg/mL | Sigma-Aldrich (A7030-10g) |
|  | DPBS | 1x | Sigma-Aldrich (D8537-500ML) |
| Complete KSFM | KSFM basal medium with  L-glutamine | 1x | Thermo Fisher Scientific  (17005) |
|  | Bovine pituitary extract (BPE) | 25 µg/mL | Thermo Fisher Scientific  (13028-014) |
|  | Epithelial growth factor (EGF) | 0.2 ng/mL | Thermo Fisher Scientific  (10450-013) |
|  | Isoproterenol (IP) | 1 µM | Sigma-Aldrich |
|  | P/S | 1x (100 U/mL Penicillin and 100 µg/mL Streptomycin) | Sigma-Aldrich (P0781) |
| Complete BEGM | BEpiCM medium | 1/2x | ScienCell (3211) |
|  | DMEM medium | 1/2x | STEMCELL Technologies (36250) |
|  | 2x BEpiCGS supplement | 1x | STEMCELL Technologies (3262) |
|  | HEPES buffer | 12.5 mM | Gibco, Thermo Fisher Scientific (15630-056) |
| 10x Soft Trypsin | Difco Trypsin 1:250 | 0.3% | BD Biosciences (215240– 100g) |
|  | EDTA | 0.1% | Sigma-Aldrich (E1644-1KG) |
|  | D-(+)- Glucose | 1% | Sigma-Aldrich (G6152-100G) |
|  | DPBS | 1x | Sigma-Aldrich (D8537-500ML) |
| Soy Bean Trypsin Inhibitor (SBTI) | SBTI | 1.1 mg/mL | Sigma-Aldrich (T9128-1G) |
|  | KSFM basal medium with L-glutamine | 1x | Thermo Fisher Scientific (17005) |
|  | P/S | 1x (100 U/mL Penicillin and 100 µg/mL Streptomycin) | Sigma-Aldrich (P0781) |

**Supplementary Table 2: Used primary and secondary antibodies**

| **Primary antibody** | **Dilution** | **Supplier** | **Cat. No.** |
| --- | --- | --- | --- |
| FOXJ1, goat polyclonal | 1:100 | R&D Systems | AF3619 |
| MUC5AC, mouse monoclonal | 1:500 | Abcam | ab3649 |
| KRT5, rabbit polyclonal | 1:500 | ITK diagnostics BV, BioLegend | 905501 |
| **Secondary antibody** |  |  |  |
| Alexa Fluor 488 Donkey anti Mouse IgG | 1:500 | Jackson ImmunoResearch | 715-545-151 |
| Alexa Fluor 594 Donkey anti Goat IgG | 1:500 | Jackson ImmunoResearch | 705-585-147 |
| Alexa Fluor 647 Donkey anti Rabbit IgG | 1:500 | Jackson ImmunoResearch | 711-605-152 |

**Supplementary Table 3: Used primers RT-qPCR**

| **Primer name** | **Primer sequence** |
| --- | --- |
| HCoV-NL63-F | ACGCAATGCCACTGTTGTTA |
| HCoV-NL63-R | GACAACACCGTCATCAGAGA |
| IRF9-F | CCACCGAAGTTCCAGGTAACAC |
| IRF9-R | AGTCTGCTCCAGCAAGTATCGG |
| STAT1-F | ATGGCAGTCTGGCGGCTGAATT |
| STAT1-R | CCAAACCAGGCTGGCACAATTG |
| ISG15-F | CTCTGAGCATCCTGGTGAGGAA |
| ISG15-R | AAGGTCAGCCAGAACAGGTCGT |
| IFIT1-F | GCCTTGCTGAAGTGTGGAGGAA |
| IFIT1-R | ATCCAGGCGATAGGCAGAGATC |
| MX1-F | GGCTGTTTACCAGACTCCGACA |
| MX1-R | CACAAAGCCTGGCAGCTCTCTA |
| IRF1-F | GAGGAGGTGAAAGACCAGAGCA |
| IRF1-R | TAGCATCTCGGCTGGACTTCGA |

**Supplementary Table 4 – Troubleshooting**

| **Problem:** | **Potential solution** |
| --- | --- |
| Leakage of medium from basolateral compartment to apical compartment | This is a critical failure. If media fills upper chamber ALI is not fully supported. Chips nee to be discarded. |
| Medium evaporation in the chip | Fill the outer part of chip within petri, surrounding the PDMS. Use a solution of PBS with 1% P/S that will increase local humidity. |
| Bubbles appear during seeding on the chip | Try to reinject the cell seeding solution slowly while keeping the outlet pointing upwards. The bubbles will moveupwards towards the oulet. |
| Cells detach from a membrane. | Use a adequate membrane for culturing each primary cells. PBECs used in this study showed affinity to PET and found difficulties attaching to the PC membranes, with poor adhesion in confluent monolayers and at times resulting in long stretched cells. Screen for materials compatibility prior to scale-up production. |
| Poor cell adhesion (at seeding) | If you are already using a selected adequate membrane and culture issue persist, evaluate the quality of the protein coating. Ensure the coating solution is incubated on the membrane for a minimum of 2 hours at 37°C to allow for sufficient protein adsorption. Ensure that your coating was 2h+ on the membrane at 37 degrees. Optimize the coating components (e.g., protein type and concentration) to meet the specific adhesion requirements of your cell type. |
| Epithelial cells do not show cornerstone pattern, showing an elongated morphology | Achieving a proper seeding density is critical for uniform and fast packing of cell into the cornerstone pattern. Success depends on both the cell suspension concentration and the delivery volume. Ensure the seeding volume is large enough to perfuse the entire microfluidic network, which delivers an adequate number of cells to the central chamber. For optimal results, use a cell suspension of 4–5 x 10⁶ cells/mL, using at least 20 µl of injected volume. Refreshing the medium 3x per week will be important to keep enough nutrition. |
| Bacterial contamination | Ensure that the all material is sterilized and include regular cleaning procedures prevent contaminations. Use clean tips with every new chip seeding, especially do not reuse tips if touched by PDMS parts. When finding difficulties getting UV proper sterilization, consider using 70% EtOH washing and drying overnight within laminar flow cabinet. |
